# Commentary: Risky decision-making is associated with residential choice in healthy older adults

**DOI:** 10.3389/fpsyg.2016.01304

**Published:** 2016-08-31

**Authors:** Dafina Petrova, Rocio Garcia-Retamero

**Affiliations:** ^1^Department of Experimental Psychology, Mind, Brain, and Behavior Research Center, University of GranadaGranada, Spain; ^2^Max Planck Institute for Human DevelopmentBerlin, Germany

**Keywords:** risk taking, ageing, older adults, balloon analog risk task (BART), motivation

Seaman et al. (2015) compared 23 older adults living independently to 23 older adults living in a retirement community on their risky decision making. Results from the Balloon Analog Risk Task (BART; Lejuez et al., 2002) showed that older adults living in the retirement community were more risk-averse, at least partially due to higher initial perceptions of risk (Seaman et al., 2015). Drawing on findings from the literature on older adults' experiences with age-related relocation and theories on aging and decision making, we suggest that a focus on cognitive factors alone may be insufficient to understand the aging decision maker. Rather, risky decision making in aging could be best understood from an integrated perspective, considering cognitive, social, and motivational factors alike.

Seaman et al. (2015) mostly conceptualize the resulting living arrangements of participants as a choice, suggesting that one possible explanation of the results is that more risk-averse older adults choose the security of a retirement community. However, research shows that older adults can be pressured into assisted living by their relatives or by life circumstances (Sergeant and Ekerdt, 2008). For instance, the majority of older adults strongly prefer to continue living in their own homes as long as possible (e.g., more than 80% of United States individuals older than 65, Wylde, 2008). If they need help caring for themselves, most respondents prefer not to move from home, and only a minority (9%) prefers to move to a facility where care is provided (Bayer and Harper, 2000). While some older adults may have a desire to move to a better home environment (Hillcoat-Nalletamby and Ogg, 2014), research shows that relocation to a retirement community or institution often occurs as a consequence of impactful life events like the death of a spouse or critical health-related events like hospitalizations or acute illness (Hays, 2002; Lee et al., 2002; Pope and Kang, 2010).

Moving to a residential care facility is mostly perceived as a stressful, negative experience by older adults, associated to feelings of loss, and suffering (Lee et al., 2002). Stress, in its acute or chronic form, can have profound and complex effects on decision making under risk, especially among older adults (Mather et al., 2009; Starcke and Brand, 2012; Weller et al., 2014). Whether, stress is disadvantageous can depend on the context and its effects can be different for men and women (Weller et al., 2014). For instance, acute stress increased risk aversion and decreased performance among older but not younger adults in a task very similar to the BART (Mather et al., 2009). Hence, as discussed by the authors, it is also possible that the challenges of adjusting to life in a retirement community may have made people more risk averse.

What are then the mechanisms that can explain this increased risk aversion? Theory and research suggest that risk aversion may reflect older people's motivation-based strategy to cope with the demanding circumstances, be it of life decisions or laboratory tasks. To explain this argument further, we turn to several studies that used the BART to investigate age differences in risk taking. Koscielniak et al. (2016) found that, compared to younger adults, older adults were more risk averse and performed worse on the BART. Rolison et al. (2012) found that compared to younger adults, older adults had higher risk perceptions, and were less certain about their risk taking. These latter results were replicated by Seaman et al. (2015) in their sample of older adults covering a much narrower age range. Taken together, increased risk aversion with age is observed in the BART. Interestingly, controlling for age and cognitive function, older adults living in a retirement community are even more risk-averse than older adults living independently. In other words, despite equal chronological age, retirement community residents display a pattern consistent with that of older age that may have to do with the relocation-related experiences discussed previously.

Research shows that older adults manage with their deteriorating cognitive processing through selective allocation of cognitive resources (Hess, 2014). For instance, if costs are perceived as high, tasks are not personally relevant, and confidence is low, older adults will limit their cognitive effort and may be less motivated to engage with decisions (Ennis et al., 2013; Hess, 2014; Bruine de Bruin et al., 2015; Strough et al., 2015). In particular, prominent theories of aging refer to allocation of resources to more positive experiences (e.g., Labouvie-Vief, 2003; Carstensen, 2006). According to socio emotional selectivity theory, older adults increasingly recognize that they have limited time left to live, so they become more motivated to increase their positive and meaningful emotional experiences and less motivated to expand their horizons (Carstensen, 2006). For example, older adults attend to and remember positive information more often (Carstensen, 2006). In their goals, they may be less oriented toward gain and growth (e.g., earning points in the BART; exploring the limits of the task by engaging in risk taking), and more oriented toward maintenance and loss prevention (e.g., cashing out and avoiding explosions, Ebner et al., 2006). Consistent with this proposition, the effect of age on risk taking in the BART is not only explained by cognitive factors (e.g., lower processing speed) but also by motivational factors (e.g., more desire for predictability and preference for order and structure, Koscielniak et al., 2016).

To translate this back to the context of Seaman et al.'s study (2015), old adults living in a retirement community display a pattern of behavior consistent with increased chronological age, potentially stronger perceptions of depleted resources, and motivation to avoid negative experiences. Longitudinal research can disentangle how these potential mediators relate to residential circumstances and increased risk aversion. We would like to encourage researchers to build on this work by considering the complexity of multi-domain decisions like residential choice. Such decisions may be better predicted by adopting an integrated multilevel approach, such that relevant cultural, emotional or social circumstances are taken into account in combination with traditional cognitive factors studied in aging.

## Author contributions

All authors listed, have made substantial, direct and intellectual contribution to the work, and approved it for publication.

### Conflict of interest statement

The authors declare that the research was conducted in the absence of any commercial or financial relationships that could be construed as a potential conflict of interest.
